# PDBsum additions

**DOI:** 10.1093/nar/gkt940

**Published:** 2013-10-22

**Authors:** Tjaart A. P. de Beer, Karel Berka, Janet M. Thornton, Roman A. Laskowski

**Affiliations:** ^1^European Molecular Biology Laboratory, European Bioinformatics Institute (EMBL-EBI), Wellcome Trust Genome Campus, Hinxton, Cambridge CB10 1SD, UK and ^2^Department of Physical Chemistry, Regional Centre of Advanced Technologies and Materials, Faculty of Science, Palacký University Olomouc, tř. 17. listopadu 12, 771 46 Olomouc, Czech Republic

## Abstract

PDBsum, http://www.ebi.ac.uk/pdbsum, is a website providing numerous pictorial analyses of each entry in the Protein Data Bank. It portrays the structural features of all proteins, DNA and ligands in the entry, as well as depicting the interactions between them. The latest features, described here, include annotation of human protein sequences with their naturally occurring amino acid variants, dynamic graphs showing the relationships between related protein domain architectures, analyses of ligand binding clusters across different experimental determinations of the same protein, analyses of tunnels in proteins and new search options.

## INTRODUCTION

PDBsum is a web server that provides overviews and pictorial analyses of the experimentally determined models of macromolecular structures in the Protein Data Bank (PDB) (1). It depicts the molecules in each entry and shows schematically the interactions between them. As of September 2013, there are >93 000 structures available. Each has its own set of PDBsum pages that provide the various analyses and data. Links to references and citations of the key reference are also provided.

PDBsum has been available since 1997 (2), with various improvements made since (3–5). Here we describe the recent additions and expansions made to PDBsum since 2009 (6).

## HUMAN VARIATION DATA

With the release of the 1000 Genomes project data (7), a large number of natural human variants at the DNA level have been identified. Variants occurring in protein-coding regions can alter the corresponding amino acids in the protein’s sequence and consequently affect the structure and/or function of the protein itself. Knowledge of the structural context of any variant can provide a better understanding of its effects. Thus, for all the human protein structures in the PDB, we have mapped all single amino acid variants from the 1000 Genomes project to the corresponding protein sequences. We used the mappings in UniProtKB (8) between the Ensembl transcript (9) and the UniProt identifier before identifying the corresponding position in the protein structure via SIFTS (10).

**Figure 1.**
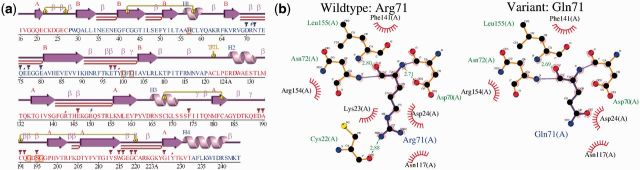
Natural human variants for human coagulation factor Xa mapped onto the 3D structure of the protein in PDB entry 2p16. (**a**) The protein’s ‘wiring diagram’ is annotated with small lightning bolt images to show residues that, according to the 1000 Genomes data, have natural variants (R71 and R150 in this case). The colouring of the protein’s sequence represents the two structural domains, whereas the purple lines, helices and arrows depict the secondary structures: coil, α-helix and β-strand, respectively. The UniProtKB, Pfam and the Ensembl gene identifier references are given above the diagram and the full set of variants listed to the left (not shown). In (**b**) are shown two LigPlot diagrams of the interactions of the wild-type and the mutated residue with the surrounding protein residues. The left diagram shows the interactions made by the wild-type residue, Arg71, and the right diagram shows those of the variant, Gln71. The residue of interest is drawn with purple bonds, the interacting residues being drawn in orange. Hydrogen bonds are represented by green dotted lines and residues interacting via non-bonded contact are shown as red eyelashes.

The variants are shown on each human structure’s ‘protein page’ in PDBsum, their location in the sequence being identified on the ‘wiring diagram’ by a small lightning flash symbol (Figure 1a). A list of the protein’s variants is also provided, with cross-references to the relevant UniProt identifier, dbSNP identifier (11) and Ensembl gene and transcript identifiers given for each.

To provide the structural context of each variant, we have used Modeller 9v3 (12) to automatically generate a homology model of the protein with variant residue, based on the wild-type structure. The model is shown in Jmol (http://www.jmol.org/) on the variant’s page and used to generate a LigPlot diagram [Figure 1b, (13)] of the interactions the variant residue makes with its surroundings (generally neighbouring residues, but occasionally bound ligands or metals). A similar LigPlot diagram of the wild-type structure is shown for comparison.

## DOMAIN ARCHITECTURE NETWORKS

Proteins can be described in terms of sequence or structural domains, with the same domains cropping up in different combinations in different proteins. PDBsum uses Pfam’s (14) sequence domain definitions and structural domains as defined by CATH (15). Each PDBsum entry shows a little schematic diagram depicting each protein’s Pfam and CATH domains. Also shown is the ‘structural coverage’ provided by the PDB entry—that is, how much of the protein is present in the 3D structure. Thus, one can tell at a glance whether a given entry is of the entire protein or only of, say, one of the domains.

Two new additions have been made to extend the usefulness of this diagram. The first is that each of the Pfam domains in the diagram is hyperlinked to a list of all other PDB entries containing that domain. The list is organized by ‘domain architecture’ (i.e. sequence of Pfam domains making up each protein). This may be useful if, say, the protein you are studying is missing a domain of interest; the list may lead you to PDB entries in which this domain is present.

**Figure 2.**
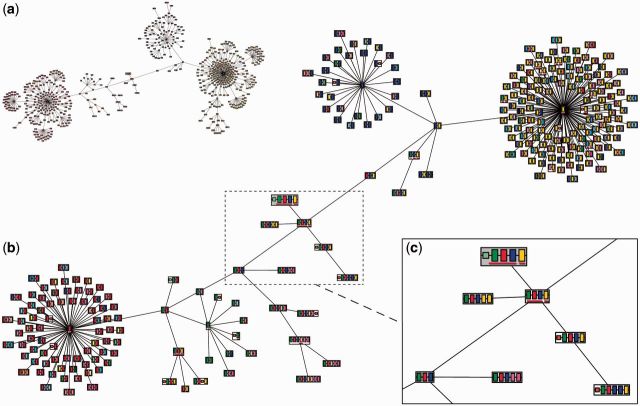
An ArchSchema domain architecture network for coagulation factor Xa from *Homo sapiens*, PDB entry 2p16. (**a**) The initial network shows 622 domain architectures (i.e. sequences of Pfam domains), each represented by a different node on the graph. There are over 5500 domain architecture with one of more domains in common with that of coagulation factor Xa, but the graph is automatically trimmed to show only the closest relatives. (**b**) The same graph, but trimmed via the ArchSchema controls to remove the more distant nodes. (**c**) A blow-up of the central section of the trimmed graph. Here one can see the sequences of individual Pfam domains, as depicted by the coloured boxes inside each node—taller boxes corresponding to Pfam-A domains and smaller ones to Pfam-B. The node representing coagulation factor Xa is the slightly larger box with a grey background at the top of the inset. Red lines underneath domains indicate there is at least one entry containing the domain in the PDB. (Clicking on the node and then on the proteins marked with a green tick takes you to the structures). Satellite nodes can be added to the plots by selecting the type required: UniProtKB sequence identifiers, PDB identifiers or enzyme classes.

The second addition is a link to the ArchSchema programme (16)—accessed via the triangular icon above the domain diagram. ArchSchema shows a dynamic 2D network of domain architectures that are most closely related to that of the protein in question. So, say your protein contains three Pfam domains, the diagram will display the domain architectures containing one or more of these three domains. Figure 2 shows an example network. The slightly enlarged and greyed out node corresponds to the domain architecture of the protein in question. Other nodes are linked to it according to the domains they have in common. Nodes underlined in red correspond to domains for which there is structural information in the PDB.

The network is interactive in that elements can be dragged around, even while the diagram is rearranging itself. Various types of satellite nodes can be switched on and off. These are UniProt ids, PDB codes or enzyme classes (i.e. E.C. numbers) associated with each architecture. Various filters are available, including selection by species, inclusion of nodes with PDB entries only and inclusion of ‘reviewed’ UniProt sequences only (i.e. Swiss-Prot sequence entries).

Similar diagrams showing domain architectures for CATH domains can be generated from each PDBsum entry’s ‘protein page’.

## LIGAND CLUSTER ANALYSIS

The PDB contains many structural determinations of the same proteins: some solved by different research groups at different resolutions, some spanning different parts of the protein sequence, some of apo structures and others having different ligands bound. One new feature in PDBsum is a ‘ligand cluster analysis’ for such proteins that specifically highlights binding sites and how different molecules bind to them.

The ligand cluster analysis is accessed via the small blue and green icon, showing two superposed ligands, above the domain diagram on the relevant PDBsum entry’s page. The clusters for each protein are precomputed as follows.

First, PDBsum chooses one entry as the ‘reference’. This will be the one having the greatest similarity to the corresponding UniProt sequence and the highest resolution and R-factor. All other structures of the same UniProt identifier are then superposed on this reference, bringing any bound ligands with them. The net result is a superposition of the binding sites and the molecules bound to them, which appear as clusters of ligands on the surface of the reference structure. The largest cluster tends to be the protein’s active site, but other clusters are also often present. In some cases these may be allosteric binding sites, in other cases just locations where small molecules such as sulphates or crystallization buffer molecules have bound.

The separate ligand clusters are colour-coded and the ligands in each cluster are listed with links to the PDB entries in which they are found. Jmol and Rasmol (17) views can show either individual ligand clusters or the reference structure with all clusters superposed. An SDF file of the ligands in each cluster can be downloaded.

Complications arise when the set of structures for a protein do not overlap in terms of sequence—for example, one group are of one domain and another group of a different domain. PDBsum shows these ‘groups’ separately, performing the ligand analysis independently for each.

## DRUGPORT

The PDB contains many structures of interest for structure-based drug design—either because the structures are of proteins that may be drug targets, or because they contain a known drug molecule bound to protein or DNA, or, in the best case, because they show a drug bound to its target. One branch of PDBsum, called DrugPort (http://www.ebi.ac.uk/thornton-srv/databases/drugport/), focuses on the drug molecules and targets that are in the PDB.

The information on drugs and drug targets is obtained from the DrugBank database (18). Drug molecules in the PDB are identified by matching the SMILES strings given in DrugBank to those in the wwPDB’s Chemical Component Dictionary. Drug targets are identified via their UniProt identifier.

**Figure 3.**
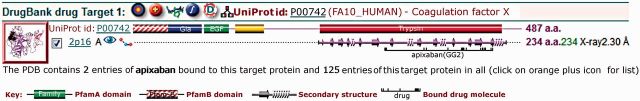
The target summary provided by DrugPort for the drug apixaban, showing its protein target, coagulation factor Xa. The table shows the UniProt identifier and a schematic diagram of the protein’s Pfam domains (as coloured cylinders). The PDB entries of the protein are represented by the purple schematic diagrams of their secondary structure (the spring-like regions representing α-helices and the arrows corresponding to β-strands). These give an idea of the structural coverage given by the PDB entry and, in this case, one can clearly see that only the trypsin domain has been solved. The red border round the thumbnail for Target 1 indicates that the PDB structures are of the drug molecule bound to that target. The schematic diagrams indicate the residue positions in the protein where the drug molecule binds. Various links are given by the little row of icons in the table headers, including a link to an ArchSchema network (Figure 2) and a ligand cluster analysis (the blue and green superposed molecules) described in the main text.

As of September 2013, there were 460 molecules (405 drugs and 55 nutraceuticals) in DrugPort. Although DrugBank had 1682 approved drugs and nutraceuticals at the time, the majority of them were not found in the PDB. For every drug in DrugPort, the molecular structure is shown as well as the number of targets (as defined by DrugBank), the number of these targets in the PDB and, more interestingly, the number that have the drug molecule bound. Figure 3 shows an example entry for the drug apixaban. The drug targets human coagulation factor Xa. For the target, there were two entries in the PDB containing the drug bound to its target and 123 entries without the drug bound (September 2013). Only part of the protein had been crystallized and fortunately the drug binds to this trypsin-like domain.

For the target, the domain diagram shows the Pfam domain architecture of the target protein and the structural coverage provided by the PDB entries. Above the diagram are several icons, including the ArchSchema and ligand cluster icons described earlier.

## PROTEIN TUNNELS

Another new analysis displays all the ‘tunnels’ in a given protein structure. These are openings that penetrate into the protein’s interior. The analysis covers the entire structure and is accessed via the Tunnels tab at the top of each PDBsum entry.

**Figure 4.**
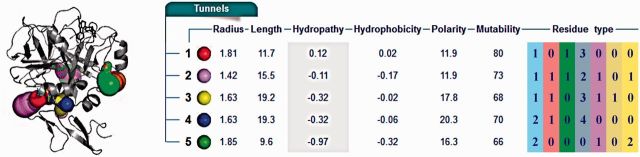
The tunnels calculated for PDB entry 2p16, excluding ligands. The tunnels are the coloured shapes embedded in the grey secondary structure representation of the protein. The table on the right shows various properties for each tunnel, the colours of the balls in the table corresponding to the colours in the image.

The tunnels are computed using Mole 2.0 (19). Two sets are calculated with and without ligands (Figure 4). First, the Voronoi diagram of the atomic centres of the protein is calculated. Then, any cavities are identified and their start and end points linked. Various tunnel properties are computed and tabulated according to the amino acids lining the tunnel. Ligands present within the tunnels are listed. An animated GIF shows a spinning representation of the tunnel locations and a link is given to the MOLEonline2.0 website (20) for more detailed analyses with user-defined tunnel searches. Large structures (i.e. having >4 protein chains) are omitted as they are currently too computationally intensive to calculate.

### NEW SEARCHES

Three new searches have been added to the PDBsum home page, including searches by UniProt accession number, Pfam domain name and Ensembl gene or transcript identifier. In each case, the results provide a list of structures matching the search term as well as the structural coverage that each structure has in comparison with the UniProt sequence.

## CONCLUSION

The new additions to the PDBsum website offer its users additional opportunities to explore protein structures and their variation, to visualize protein domain architectures and to analyse ligand–protein interactions by means of ligand-binding cluster analysis and protein tunnel characterization.

## FUNDING

National Institutes of Health [GM094585], by the U. S. Department of Energy, Office of Biological and Environmental Research, under contract DE-AC02-06CH11357 (Midwest Center for Structural Genomics), with Federal funds from the National Institute of Allergy and Infectious Diseases, National Institutes of Health, Department of Health and Human Services (in part), under Contracts No. HHSN272200700058C and HHSN272201200026C. The authors would also like to thank EMBL-EBI for providing the computational resources and support (for T.d.B). K.B. acknowledges support from the Czech Science Foundation (project P208/12/G016) and the Operational Program Research and Development for Innovations—European Regional Development Fund [CZ.1.05/2.1.00/03.0058] and the European Social Fund [CZ.1.07/2.3.00/20.0058]. Funding for open access charge: EMBL-EBI.

*Conflict of interest statement*. None declared.
